# Supplementary material for: Vocational rehabilitation for people with multiple sclerosis: A systematic scoping review of international evidence
Source: PLoS One. 2026 May 27;21(5):e0350122. doi: 10.1371/journal.pone.0350122 (PMC13215546; doi:10.1371/journal.pone.0350122)
Supplement: S1 Table — Search strings used for each electronic database, including MeSH terms and free-text keywords. (DOCX) [file pone.0350122.s001.docx]

### Research Query in PubMed

| Domain | Query |
| --- | --- |
| #1 Population | "Demyelinating Autoimmune Diseases, CNS"[Mesh] OR “multiple sclerosis”[Tiab] OR “MS”[Tiab] OR “demyelinating autoimmune diseases”[Tiab] OR “demyelinating autoimmune disorders”[Tiab] OR “clinically isolated syndrome”[Tiab] OR “demyelinating”[Tiab] |
| #2 Concept | "Rehabilitation, Vocational"[Mesh] OR "Return to Work"[Mesh] OR “vocational guidance”[Mesh] OR "Employment, Supported"[Mesh] OR “prevocational training”[Tiab] OR “Job retention”[Tiab] OR “Work retention”[Tiab] OR “work rehabilitation”[Tiab] OR “work maintenance”[Tiab]OR “Job maintenance”[Tiab] OR "work adjustment"[Tiab] OR “job adjustment"[Tiab] OR “job placement” [Tiab] OR “work placement”[Tiab] OR “work hardening” [tiab] OR “work conditioning”[tiab] OR “reasonable accommodation”[Tiab] OR (“vocational*”[tiab] AND (“training”[tiab] OR “therapy”[tiab] OR “rehabilitation”[tiab] OR “intervention”[tiab] OR “service*”[tiab] OR “program*”[tiab] OR “councelling”[tiab] OR “interest*”[tiab])) OR (“Occupational”[tiab] AND (“rehabilitation”[tiab] OR “intervention”[tiab] OR “program*”[tiab])) |
