# Supplementary material for: Vocational rehabilitation for people with multiple sclerosis: A systematic scoping review of international evidence
Source: PLoS One. 2026 May 27;21(5):e0350122. doi: 10.1371/journal.pone.0350122 (PMC13215546; doi:10.1371/journal.pone.0350122)
Supplement: S2 Table — Detailed characteristics of participants across the included studies, including, geographical origins, marital and occupational status, MS type, EDSS and disease duration. (DOCX) [file pone.0350122.s002.docx]

| **Article** | **year** | **Origins** | **Level of education** | **Marital status** | **Occupational status** | **EDSS** | **MS phenotype** | **Disease duration** |
| --- | --- | --- | --- | --- | --- | --- | --- | --- |
| Aarts et al., | 2024 | NA | NA | NA | NA | NA | NA | NA |
| Arntzen et al., | 2023 | NR | NR | NR | Usual care: Employed = 12 (92.3%), Not employed = 1 (7.7%). Interventional: Employed = 12.0 (80.0%), 3 (20.0%) | Usual care: 1.7 (SD=1.1). Interventional: 1.8 (SD 0.9) | Usual care: PP-MS = 2 (15.4%), RR-SP = 11 (84.6%). Interventional: PP-MS = 0 (0%), RR-MS = 15 (100%) | Usual care: 12.0 (SD=11.2). Interventional: 10.4 (SD=7.8) |
| Chiu et al., | 2013 | European American = 1420 (74.0%), African American = 376 (19.6%), Native American = 23 (1.2%), Asian American = 16 (0.8%), Hispanic American = 85 (4.4%). | Less than high school = 105 (5.5%), completed high school = 574 (29.9%), Post-secondary/associate = 733 (38.2%), college degree or higher = 508 (26.5%) | NR | NR | NR | NR | NR |
| Chiu et al., | 2015 | 6,551 (75%) were European American, 1,580 (15%) were African  American, 430 (5%) were Hispanic/Latino,  80 (1%) were American Indian, and 74 (1%)  were Asian | NR | NR | unemployed  (72 %); employed (28%) | NR | NR | NR |
| De Dios Perez et al.,_a | 2025 | White British = 15 (75%), White other = 2 (10%), Black British = 2 (10%); Asian British = 1 (5%) | A Levels = 3 (15%), College = 6 (30%), Degree = 4 (20%), Postgraduate = 6 (30%), Other = 1 (5%) | In a relationship = 11 (55%), single = 6 (30%), divorced = 2 (10%), not provided = 1 (5%) | Employment type: Public = 10 (50%), Private = 8 (40%), Self-employed = 2 (10%). | 4.45 (2.33) | PP-MS = 1 (5%), SP-MS = 5 (25%), RR-MS = 14 (79%) | NA |
| De Dios Perez et al.,_b | 2025 | NA | NA | NA | NA | NA | NA | NA |
| De Dios Perez et al., | 2024 | NA | NA | NA | NA | NA | NA | NA |
| De Dios Perez et al., | 2023 | White British = 14 (93.33%), Black British Caribben = 1 (6.67%) | A Levels = 4 (26.67%), Higher National Diploma = 4 (26.7%), College = 3 (205), Degree = 3 (20%), Postgraduate = 1 (6.67%). | Single = 1 (6.67%), in a relationship = 12 (80%), divorced/separated = 2 (13.33%) | Employed = 15 (100%), Not employed = 0 (0%) | NR | SP-MS = 6 (40%), RR-MS = 9 (60%) | NR |
| Dettmer et al., | 2021 | NR | NR | NR | White collar = 86%, Blue collar = 9 (14%) | 3.8 (1.0 - 6.5) | PP-MS = 11 (17%), SP-MS = 10 (16%), RR-MS = 43 (67) | 14.7±9.5 |
| Dorstyn et al., | 2017 | NR | High school completion = 17 (18%), degree or diploma = 60 (63%), Other (eg. Trade qualification) = 14 (15%) | Married/de facto 71 (75%) Single/divorced/widowed 24 (25%) | Employed (looking for new work) = 73 (77%), not employed = 22 (23%) | NR | PP-MS = 1 (1%), SP-MS = 5 (5%), RR-MP = 84 (88%), RP-MS = 1 (1%), Unknown = 4 (4%) | NR |
| Dorstyn et al., | 2016 | NR | Some high school = 6 (20.7%), high school completion = 3 (10.3%), undergraduate degree or diploma = 14 (48.3%), postgraduate degree = 5 (17.2%), other (e.g., trade qualification) = 1 (3.4%) | Married/partnered = 20 (69.0%), single/not partnered = 9 (31.0%) | Employed (paid work) = 17 (58.6%), unemployed = 12 (41.3) | NR | PP-MS = 0 (0%), SP-MS = 2 (6.9%), RP-MS = 2 (6.9%), RR-MS = 25 (86.2%) | 8.5 |
| Dorstyn et al., | 2022 | NR | Some high school = 1 (3.5%), completed high school = 3 (10.3%), post-secondary diploma or certificate = 8 (27.6%), undergraduate degree = 10 (34.5), postgraduate degree = 7 (24.1%) | Partnered = 18 (62.1%), Not partnered = 11 (37.9%) | Employed = 28 (96.5%), unemployed = 1 (3.5%) | NR | PP-MS = 1 (3.5%), SP-MS = 17.2%, RR-MP = 23 (79.3%) | (Median) = 115 [49-180] months |
| Hartvedt et al., | 2024 | NR | NR | NR | NR | 1.73 (SD=0.99) | PP-MS = 1, RRMS = 25 | NR |
| Inge et al., | 2016 | NR | NR | NR | NR | NR | NR | NR |
| Jellie et al., | 2014 | NR | Degree = 15 (79%); A levels = 2 (7%); Secondary education = 1 (4%); Post-graduate = 1 (4%) | NR | NR | NR | PP-MS = 3 (16%), SP-MS = 2 (7%), RR-MS = 14 (74%) | NR |
| Meyer‑Moock et al., | 2022 | NA | NA | NA | NA | NA | NA | NA |
| Nornematolahi et al., | 2013 | NR | NR | NR | NR | NR | NR | 4±1 |
| Rumrill et al., | 1998 | Group 1 (n = 23). White = 83%, not white = 17%. Group 2 (n = 14). White = 79%, not white = 21% | Group 1 = 14 years; Group 2 = mean 14 years | NR | NR | NR | NR | Group 1 = 12 Group 2 = 12 |
| Rumrill et al., | 2013 | Caucasians = 35 (85%), African American = 6 (15%) | Graduate degree = 26 (63%), no graduate degree = 9 (37%) | Married = 21 (51%), not married = 20 (49%) | Employed = 24 (59%), not employed = 17 (41%) |  | NR | 14.8 (8.0) |
| Rumrill et al., | 1996 | NA | NA | NA | NA | NA | NA | NA |
| Stimmel et al., | 2020 | 25 (83.3%) white; other 5 (16.7%) | High school or less 4 (13.3%); at least some college 26 (86.7%) | 19 (63.3%) Married/cohabiting; 6 6 (20.0%) single/engaged; 3 (10.0%) divorced; 2 (6.7%) widowed | NR | NR | NR | 9.1 (SD = 8.2) |
| Stimmel et al., | 2022 | Standard-care intervention. White = 17(73.9%), afrincan american = 4 (4.3%), hispanic = 3 (13.0%), Other = 2 (8.7%). Experimental intervention. White = 24 (72.7%), African American = 2 (6.1%), Hispanic = 3 (9.1%), other = 12.2% | Standard-care intervention (n = 23). Mean = 15.1 years (SD = 2.1). Experimental intervention (n = 33) = 15.3 (1.7). | Standard-care intervention. Married/cohabiting = 14 (60.8%), single/engaged = 2 (8.7%), divorced/separated = 6 (26%), widowed = 1 (4.3%). Experimental intervention. Married/cohabiting = 19 (57.6%), single/engaged = 11 (33.3%), divorced/separated = 2 (6.1%), widowed = 1 (3.0%) | Standard-care intervention: Employed =100%. Experimental intervention: Employed = 100% | NR | NR | Standard-care intervention: 9.8 (8.3%). Experimental Intervention = 8.5 (7.9) |
| Strauser et al., | 2018 | NA | NA | NA | NA | NA | NA | NA |
| Sweetland et al., | 2014 | NR | NR | NR | Employed = 2 (100%) | NR | RR-MP = 2 (100%) | NR |
| Tansey et al., | 2015 | 1,420 (74%) European Americans, 376 (19.6%) African Americans, 85 (4.4%) Hispanic or Latino Americans, 23 (1.2%) Native Americans, and 16 (0.8%) Asian Americans. | NR | NR | NR | NR | NR | NR |
| Van der Mei et al., | 2024 | NA | NA | NA | NA | NA | NA | NA |
| Vonck et al., | 2023 | NR | NR | NR | Employed = 118 (100%) | NR | NR | NR |
| Wickstrom et al_North_a., | 2017 | NR | Incomplete primary school = 2 (1%), primary school = 10 (6%), 2-4 years secondary school = 65 (41%), university = 82 (52%) | NR | Employed = 124, not employed = 56 | Median = 2 (1.0-3.5) | PP-MS = 18 (10%), RR-PM = 127 (73%), SP-MS = 30 (17%) | 11 (7.8) |
| Wickstrom et al_South_b., | 2017 | NR | Incomplete primary school = 1 (1%), primary school = 11 (7%), 2-4 years secondary school = 79 (48%), university = 72 (44%) | NR | Employed = 109, unemployed = 79 | Median = 2(1.0-3.5) | PP-MS = 13 (8%), SP-MS = 47 (28%), RR-MS = 108 (64%). | 13 (8.7) |

Legend: NR= Not reported; NA = Not applicable
