# Supplementary material for: Vocational rehabilitation for people with multiple sclerosis: A systematic scoping review of international evidence
Source: PLoS One. 2026 May 27;21(5):e0350122. doi: 10.1371/journal.pone.0350122 (PMC13215546; doi:10.1371/journal.pone.0350122)
Supplement: Attachment — Submitted filename: Review PLOS one 01-05-2026.docx [file pone.0350122.s005.docx]

I enjoyed reading this paper and I like the scope of this review including a summary of stakeholders’ perspective. I do have some concerns, which are listed below:

- There should be **one** clear definition of vocational rehabilitation that is used throughout the article. The authors use different definitions in the abstract (‘Vocational rehabilitation aims to optimize job access and the return to work through multidisciplinary and person-centered approaches.’, ‘Studies were eligible if they were related to VR interventions for pwMS, focused on job access, return, or retention and if they were primary articles’) and introduction (‘Vocational rehabilitation (VR) aims to ease work difficulties and to optimise work participation in individuals with chronic illness or disability’)
- The systematic review from Kahn and colleagues is very relevant for this topic and should be mentioned in the article: [Effectiveness of vocational rehabilitation intervention on the return to work and employment of persons with multiple sclerosis - PubMed](https://pubmed.ncbi.nlm.nih.gov/19160331/)
- It should be clear in table 1 who the target group for the intervention is since the authors include VR interventions for people who are working, who are looking for work etc. and these groups are very different
- The descriptive statistics in the text describe which articles included different characteristics of the population (e.g. ethnicity) but the article does not specify this further; I’d like to see this either in the text or one of the tables (e.g. what ethnicities were included and in which articles)
- The authors conclude that there is a pivotal role of VR interventions in facilitating access to and retention in work for PwMS, however effects were not taken into account in this review so that statement feels out of place
