# Supplementary material for: Vocational rehabilitation for people with multiple sclerosis: A systematic scoping review of international evidence
Source: PLoS One. 2026 May 27;21(5):e0350122. doi: 10.1371/journal.pone.0350122 (PMC13215546; doi:10.1371/journal.pone.0350122)
Supplement: Attachment — Submitted filename: PLOS one_Response to reviewer.docx [file pone.0350122.s007.docx]

**Response to reviewers’letter**

**Reviewer general comment and Journal requirements**

We would like to thank the reviewer for their helpful suggestions, which we believe will improve the quality of the article. Below, we provide our responses to each of the comments raised, together with the changes made to the manuscript. The changes made can also be viewed in the version with tracked changes, titled ‘Revised Manuscript with Track Changes’.

In addition to the changes requested by the reviewer, the ‘Revised Manuscript with Track Changes’ file includes the corrections made to the reference list. No articles have been added or removed; only the numbering in the reference list has been adjusted to match the numbers used in the text for the citations. We would therefore also like to thank the editor for advising us on how to amend the manuscript. Changes relating to formatting, funding and authors’ contributions are also tracked within the version.

**Author’s Response**

**Reviewer specific comments and responses**

1. There should be **one** clear definition of vocational rehabilitation that is used throughout the article. The authors use different definitions in the abstract (‘Vocational rehabilitation aims to optimize job access and the return to work through multidisciplinary and person-centered approaches.’, ‘Studies were eligible if they were related to VR interventions for pwMS, focused on job access, return, or retention and if they were primary articles’) and introduction (‘Vocational rehabilitation (VR) aims to ease work difficulties and to optimise work participation in individuals with chronic illness or disability’)

#1: We would like to thank the reviewer once again for giving us the opportunity to clarify the theoretical background on which this scoping review is based. Throughout the scoping review, our intention was to consistently use the definition provided by Escorpizo et al (2011). To avoid any confusion regarding differing definitions, we have revised the abstract and main text to ensure consistent use of a single definition of vocational rehabilitation throughout the manuscript and clarified all relevant passages accordingly.

In the abstract, VR interventions are now described as follow:

*According to Escorpizo et al., 2011 framework, Vocational rehabilitation (VR) aims to optimise job participation, providing support in the job access, retention and in the return to work for people with disability.*

In the introduction:

*According to Escorpizo et al., 2011, Vocational rehabilitation (VR) aims to optimise job participation in individuals with disability [20], and it has emerged as a promising method for delivering employment support services to individuals with different conditions [21]. In this framework, VR is conceptualised as an intervention capable of supporting people with disabilities at all stages of their working life, facilitating access in the workforce and job retention , as well as in the return to work [20]*

In the eligibility criteria:

*In line with the definition proposed by Escorpizo et al. 2011, we included all types of VR interventions aimed at supporting pwMS in access to, retention in, and return to work.*

2. The systematic review from Kahn and colleagues is very relevant for this topic and should be mentioned in the article: [Effectiveness of vocational rehabilitation intervention on the return to work and employment of persons with multiple sclerosis - PubMed](https://pubmed.ncbi.nlm.nih.gov/19160331/)

#2: We would like to thank the reviewer for their suggestion. The Cochrane systematic review by Khan et al. (2009) has been discussed as a relevant background source in both the Introduction (74 and 101-102) and in the Discussion (453-454). It is included as Reference 3 in the reference list. To improve clarity and facilitate identification, we have checked the citation and ensured that the full reference is accurately reported in the manuscript.

In addition, we have included a more explicit reference to the review in the introduction (lines 101–102), as follows:

*However, VR for pwMS is still an emerging field [22, 23], and evidence regarding the efficacy of VR for pwMS is still limited, as highlighted in the Cochrane review by Khan et al., 2009, [3].*

3. It should be clear in table 1 who the target group for the intervention is since the authors include VR interventions for people who are working, who are looking for work etc. and these groups are very different

#3: We would like to thank the reviewer for this comment, which we believe will improve the qualitative summary of the results. We have therefore added a new column to Table 1, entitled ‘Worker characteristics’, in which we have included three possible categories: ‘Job seekers’, ‘Workers needing job retention support’ and ‘Workers returning to work’. All the changes can be seen in the version “Revised Manuscript with Track Changes”.

4. The descriptive statistics in the text describe which articles included different characteristics of the population (e.g. ethnicity) but the article does not specify this further; I’d like to see this either in the text or one of the tables (e.g. what ethnicities were included and in which articles)

#4: Thank you for this helpful comment. Detailed study-level information regarding participant sociodemographic and population characteristics (including, where reported, ethnicity/geographical origin, education, marital status, and occupational variables) is provided in the Supplementary Materials (Table S2). Given the heterogeneity in the reporting of these variables across studies, we chose to summarise the descriptive statistics narratively in the main text while presenting the full study-level details in the supplementary materials to preserve readability and avoid overburdening the main manuscript. In the narrative synthesis, we reported only the most frequently represented category within each variable (e.g., predominantly Caucasian/European participants where ethnicity was reported) to provide an overall summary of sample characteristics. To improve clarity, we have revised the manuscript to more explicitly indicate that detailed participant characteristics for each included study are available in supporting information (S2 Table. Detailed characteristics of participants across the included studies, including, geographical origins, marital and occupational status, MS type, EDSS and disease duration). All the changes can be seen in the version “Revised Manuscript with Track Changes”.

5. The authors conclude that there is a pivotal role of VR interventions in facilitating access to and retention in work for PwMS, however effects were not taken into account in this review so that statement feels out of place

#5: We would like to thank the reviewer for pointing out this inconsistency. We agree that this sentence goes beyond the findings and scope of this scoping review. We have therefore amended the conclusions, changing the sentence as follows (All the changes can be seen in the version “Revised Manuscript with Track Changes”):

*We have mapped the existing literature on VR for pwMS who need to enter, return to or remain in employment. (…)*
